# Pentafecta for Radical Nephroureterectomy in Patients with High-Risk Upper Tract Urothelial Carcinoma: A Proposal for Standardization of Quality Care Metrics

**DOI:** 10.3390/cancers14071781

**Published:** 2022-03-31

**Authors:** Frederik König, Nico C. Grossmann, Francesco Soria, David D’Andrea, Tristan Juvet, Aaron Potretzke, Hooman Djaladat, Alireza Ghoreifi, Eiji Kikuchi, Nozomi Hayakawa, Andrea Mari, Zine-Eddine Khene, Kazutoshi Fujita, Jay D. Raman, Alberto Breda, Matteo Fontana, John P. Sfakianos, John L. Pfail, Ekaterina Laukhtina, Pawel Rajwa, Maximilian Pallauf, Giovanni E. Cacciamani, Thomas van Doeveren, Joost L. Boormans, Alessandro Antonelli, Marcus Jamil, Firas Abdollah, Jeffrey Budzyn, Guillaume Ploussard, Axel Heidenreich, Siamak Daneshmand, Stephen A. Boorjian, Morgan Rouprêt, Michael Rink, Shahrokh F. Shariat, Benjamin Pradere

**Affiliations:** 1 Department of Urology, Comprehensive Cancer Center, Medical University of Vienna, 1090 Vienna, Austria; frederik.koenig1@gmail.com (F.K.); dd.dandrea@gmail.com (D.D.); katyalaukhtina@gmail.com (E.L.); pawelgrajwa@gmail.com (P.R.); maximilian.pallauf@gmail.com (M.P.); sfshariat@gmail.com (S.F.S.); 2 Department of Urology, University Medical Center Hamburg-Eppendorf, 20251 Hamburg, Germany; dr.michaelrink@gmail.com; 3 Department of Urology, University Hospital Zurich, 8091 Zurich, Switzerland; nico.grossmann@gmail.com; 4 Department of Urology, Luzerner Kantonsspital, 6004 Lucerne, Switzerland; 5 Division of Urology, Department of Surgical Sciences, San Giovanni Battista Hospital, University of Studies of Torino, 10124 Turin, Italy; soria.fra@gmail.com; 6 Department of Urology, Lions Gate Hospital, North Vancouver, BC V7L 2L7, Canada; tsjjuvet@gmail.com; 7 Department of Urology, Mayo Clinic, Rochester, MN 55905, USA; potretzke.aaron@mayo.edu (A.P.); boorjian.stephen@mayo.edu (S.A.B.); 8 Department of Urology, USC/Norris Comprehensive Cancer Center, Los Angeles, CA 90033, USA; djaladat@med.usc.edu (H.D.); alireza.ghoreifi@med.usc.edu (A.G.); daneshma@med.usc.edu (S.D.); 9 Department of Urology, St. Marianna University School of Medicine, Kawasaki 216-8511, Japan; eiji-k@kb3.so-net.ne.jp (E.K.); hoffnungen0921@gmail.com (N.H.); 10 Unit of Oncologic Minimally-Invasive Urology and Andrology, Department of Experimental and Clinical Medicine, Careggi Hospital, University of Florence, 50134 Florence, Italy; andreamari08@gmail.com; 11 Department of Urology, University of Rennes, 35000 Rennes, France; khene.zineddine@gmail.com; 12 Department of Urology, Faculty of Medicine, Kindai University, Osaka 577-8502, Japan; kazu.fujita2@gmail.com; 13 Division of Urology, Department of Surgery, College of Medicine, The Pennsylvania State University, Hershey, PA 16801, USA; jraman@pennstatehealth.psu.edu; 14 Urology Department, Fundació Puigvert, Autonomous University of Barcelona, 08193 Barcelona, Spain; albbred@gmail.com (A.B.); teo.fontana@me.com (M.F.); 15 Department of Urology, Icahn School of Medicine at Mount Sinai, New York, NY 10029, USA; john.sfakianos@mountsinai.org (J.P.S.); john.pfail@icahn.mssm.edu (J.L.P.); 16 Institute for Urology and Reproductive Health, Sechenov University, 119048 Moscow, Russia; 17 Department of Urology, Medical University of Silesia, 41-808 Zabrze, Poland; 18 Department of Urology, Paracelsus Medical University Salzburg, University Hospital Salzburg, 5020 Salzburg, Austria; 19 USC Institute of Urology, Keck Medicine of USC, University of Southern California, Los Angeles, CA 90007, USA; giovanni.cacciamani@gmail.com; 20 Department of Urology, Erasmus MC Cancer Institute, University Medical Center, 3015 GD Rotterdam, The Netherlands; t.vandoeveren.1@erasmusmc.nl (T.v.D.); j.boormans@erasmusmc.nl (J.L.B.); 21 Department of Urology, Azienda Ospedaliera Universitaria Integrata of Verona, University of Verona, 37129 Verona, Italy; alessandro_antonelli@me.com; 22 Vattikuti Urology Institute, Henry Ford Hospital, Detroit, MI 48202, USA; mjamil1@hfhs.org (M.J.); firas.abdollah@gmail.com (F.A.); jbudzyn1@hfhs.org (J.B.); 23 Department of Urology, La Croix du Sud Hospital, 31130 Toulouse, France; g.ploussard@gmail.com; 24 Department of Urology, Uro-Oncology, Robot Assisted and Specialized Urologic Surgery, University Hospital Cologne, 50937 Cologne, Germany; axel.heidenreich@uk-koeln.de; 25 Urology Department, GRC n°5, Predictive Onco-Uro, AP-HP, Pitié-Salpêtrière Hospital, Sorbonne University, 75006 Paris, France; mroupret@gmail.com; 26 Hourani Center for Applied Scientific Research, Al-Ahliyya Amman University, Amman 19328, Jordan; 27 Karl Landsteiner Institute of Urology and Andrology, 1010 Vienna, Austria; 28 Department of Urology, Weill Cornell Medical College, New York, NY 10021, USA; 29 Department of Urology, University of Texas Southwestern, Dallas, TX 75390, USA; 30 Department of Urology, Second Faculty of Medicine, Charles University, 11638 Prague, Czech Republic

**Keywords:** nephroureterectomy, pentafecta, quality, upper tract urothelial carcinoma, UTUC

## Abstract

**Simple Summary:**

Measuring the quality of care is important in health care to improve the treatment of patients. In this investigation, we sought to identify five indicators (“pentafecta”) that reflect the quality of care of patients who have cancer of the upper urinary tract (kidney and/or ureter) and are treated with surgical removal of the affected kidney and ureter. Furthermore, we searched for conditions that can predict a failure to achieve these criteria during treatment. The five indicators that define the pentafecta are the complete removal of the tumor without residuals, the complete removal of the ureter and its bladder part, the absence of complications related to the blood, the absence of severe complications related to the surgery, and the absence of tumor recurrence 12 months after the surgery. Of the 1718 patients included, 844 (49%) achieved all pentafecta criteria. These patients had higher chances at 5 years after the surgery to be alive and not to die from any cause (A) or from cancer (B) compared to those who did not achieve the pentafecta criteria (A: 68.7 vs. 50.1% and B: 79.8 vs. 62.7%, respectively). There were no conditions related to the patient that were found to predict a failure to achieve the pentafecta. Using quality indicators such as the proposed pentafecta for the assessment of the treatment of cancer patients may help define prognosis and improve patient care.

**Abstract:**

Background: Measuring quality of care indicators is important for clinicians and decision making in health care to improve patient outcomes. Objective: The primary objective was to identify quality of care indicators for patients with upper tract urothelial carcinoma (UTUC) and to validate these in an international cohort treated with radical nephroureterectomy (RNU). The secondary objective was to assess the factors associated with failure to validate the pentafecta. Design: We performed a retrospective multicenter study of patients treated with RNU for EAU high-risk (HR) UTUC. Outcome measurements and statistical analysis: Five quality indicators were consensually approved, including a negative surgical margin, a complete bladder-cuff resection, the absence of hematological complications, the absence of major complications, and the absence of a 12-month postoperative recurrence. After multiple imputations and propensity-score matching, log-rank tests and a Cox regression were used to assess the survival outcomes. Logistic regression analyses assessed predictors for pentafecta failure. Results: Among the 1718 included patients, 844 (49%) achieved the pentafecta. The median follow-up was 31 months. Patients who achieved the pentafecta had superior 5-year overall- (OS) and cancer-specific survival (CSS) compared to those who did not (68.7 vs. 50.1% and 79.8 vs. 62.7%, respectively, all *p* < 0.001). On multivariable analyses, achieving the pentafecta was associated with improved recurrence-free survival (RFS), CSS, and OS. No preoperative clinical factors predicted a failure to validate the pentafecta. Conclusions: Establishing quality indicators for UTUC may help define prognosis and improve patient care. We propose a pentafecta quality criteria in RNU patients. Approximately half of the patients evaluated herein reached this endpoint, which in turn was independently associated with survival outcomes. Extended validation is needed.

## 1. Introduction

Upper tract urothelial carcinoma (UTUC) is a rare malignancy with an estimated annual incidence of two cases per 100,000 inhabitants in western countries [1,2]. Based on preoperative characteristics, localized UTUCs are classified as low- or high-risk (HR) according to the risk of progression [3,4,5]. The optimal management of UTUC remains challenging due to its rarity and the therapeutic challenges inherent to the disease [6,7,8]. Patients treated with radical nephroureterectomy (RNU), for example, have a large variance in care delivery with the optimal management strategy and risk stratification for adjuvant therapy remaining elusive [3,9].

Quality of care assessment is becoming increasingly important. There is interest in assessing the degree in which the volume of health service may affect the likelihood of achieving quality metrics.

In urologic-oncology surgery, such criteria have been identified for various entities, using combined criteria called trifecta and/or pentafecta for partial nephrectomy, radical prostatectomy, and radical cystectomy [10,11,12,13,14]. In UTUC, on the other hand, although multiple nomograms have been proposed to improve patient selection for the best treatment [4,9,15,16,17,18,19], no defined quality of care indicators have been identified to be associated with outcomes. Predefined criteria that serve as evidence-based surrogate factors of patient-centered outcomes may help set a metric for care delivery, thereby helping to guide treatment allocation, quality assurance, and efficiency.

In this study, we aimed to propose and assess the prognostic validity of a pentafecta, identified based on evidence from the literature, in a large, multi-center cohort of patients treated with RNU for HR-UTUC.

## 2. Material and Methods

### 2.1. Cohort Description

We performed an international, multicenter, retrospective, observational study involving 21 institutions. IRB approval was given at each institution. We included patients with UTUC classified as high-risk according to the EAU guidelines [3] and treated with RNU between April 1990 and March 2020. The definition of high-risk included having any of the following: multifocal disease, tumor size ≥2 cm, high-grade cytology, high-grade biopsy, local invasion on CT, hydronephrosis, or variant histology. Patients with low-risk UTUC or distant metastasis (cM1) at the time of surgery were excluded. Patient information was collected from the medical records, and all data were anonymized before data sharing.

### 2.2. Pentafecta Criteria

Based on a comprehensive review of the literature and a consensus achieved among the co-authors (see Supplementary File S1), we identified five criteria (pentafecta) that reflected the whole perioperative management of patients with HR-UTUC. They covered three different domains: 1. two surgical aspects, the performance of an en bloc bladder-cuff excision (due to heterogeneity in the final pathological specimen report, we used only the surgeon’s surgical report) and the absence of positive surgical margins (either ureteral or soft tissue); 2. two perioperative outcomes, the absence of hematologic complications (defined as the need for perioperative transfusion and/or thromboembolic complication, such as pulmonary embolism or deep vein thrombosis) and the absence of any major complications (defined as grade ≥3 according to the Clavien–Dindo classification) within the first three postoperative months; 3. one oncological outcome, the absence of disease recurrence of any type (local, contralateral, distant, or bladder recurrence) within the first 12 months (early RFS). Indeed, the use of an oncological outcome in such tools has already been proposed in other surgeries [11,12], and early RFS has the potential to reflect the perioperative oncological management (improved RFS with accurate surgical indications, lymph node dissection, perioperative systemic therapies, and perioperative bladder instillations) [20,21,22]. When patients fulfilled all these criteria, the pentafecta was considered achieved.

### 2.3. Treatment-Specific Features and Follow-Up

The decision to perform open, laparoscopic, or robot-assisted RNU as well as to perform an LND and the extent of LND when performed [23,24,25,26,27] was at the surgeon’s discretion, based on the patient and preoperative disease characteristics. Site-specific dedicated uro-pathologists performed the examination of all surgical specimens, with tumor grade mainly being determined according to the 2016 World Health Organization (WHO) classification and tumor stage being attributed according to the 2002 Union for International Cancer Control tumor, node, and metastasis classification system (TNM) [28,29].

Regarding oncological outcomes, the pathological tumor stage and grade (pTNM); the presence of positive surgical margins on the ureter, bladder cuff, or surrounding soft tissue; the occurrence of local, contralateral, distant, or intravesical recurrence; and the administration of adjuvant chemo- or radiotherapy were assessed. The non-urothelial recurrence-free survival (NURFS) was defined by any local or distant recurrence and metastasis that occurred after RNU. Bladder recurrence was considered separately to calculate the bladder recurrence-free survival (BRFS) [8]. The overall survival (OS) was calculated as the interval from RNU to death. Patients were censored at their last follow-up. The follow-up schedule was based on the international recommendations at the time [3,30] and the physician’s preferences. In general, it included cross-sectional imaging and cystoscopy every three months for the first two years, then every six months for the second to the fifth year, then annually or six months initially, then annually.

### 2.4. Study Outcomes

The primary endpoint of this study was to assess the prevalence and prognostic value of the pentafecta among an international multicentric cohort. The secondary endpoint was to identify predictors of a pentafecta failure.

### 2.5. Statistical Analysis

Continuous variables were reported as means, medians, and interquartile ranges (IQR). To assess the differences of categorical variables between groups, the Pearson’s chi-squared or Fisher’s exact test were used, and for continuous variables, the Wilcoxon rank-sum test was used. Due to inherent imbalances in baseline patients and disease characteristics, further statistical analyses were performed in a stepwise fashion. First, to account for missing baseline data, which were assumed to be missing at random, multiple imputations using chained equations were performed. Fifteen imputed datasets were generated using predictive mean matching for numeric variables, logistic regression for binary variables, and Bayesian polytomous regression for factor variables [31]. Second, a 1:1 propensity-score-matched (PSM) analysis was performed to reduce bias and adjust for the effects of the covariate imbalances. Variables that were adjusted included age, sex, ASA, BMI, ECOG, smoking status, diabetes mellitus, hypertension, preoperative kidney function, neoadjuvant chemotherapy (NAC), CT stage, clinical lymph node enlargement, previous bladder cancer, surgical approach, performance of LND, tumor multifocality, pathological tumor stage and grade, lymph node involvement, lymphovascular invasion, concomitant carcinoma in situ, and perioperative intravesical instillation. Visual inspection using loess regression analyses and t-tests of the differences between means and standardized mean differences were used to examine the post-matching balance in covariates (see Figure S1A–D).

Third, we performed all further analyses unmatched and PSM-adjusted. Kaplan–Meier curves and log-rank tests were used to graphically visualize and compare survival outcomes (OS, CSS, NURFS, and BRFS) between the groups. Unmatched and PSM-adjusted univariable and multivariable Cox proportional hazard regression analyses were performed to estimate the hazard ratios (HR) with their 95% confidence intervals (CI) for the association with OS, CSS, and NURFS after adjusting for the effects of the relevant covariates. The evaluation of predictors for the pentafecta was assessed using PSM-adjusted univariable logistic regression models. The discriminations of the multivariable models were evaluated using Harrel’s concordance index (C-index). Two-sided statistical significance was defined as a *p* value < 0.05. All statistical analyses were performed using R (Version 4.0.3, R Foundation for Statistical Computing, Vienna, Austria, 2020).

## 3. Results

### 3.1. Baseline Clinicopathological Characteristics

Of the 2434 patients in our multi-institutional cohort, 1718 met the inclusion criteria for study here. Among them, 844 (49%) had all the pentafecta criteria, while 874 (51%) did not. After PSM adjustment, the cohort comprised 1016 patients, with 508 in each arm.

Table 1 presents the patients’ baseline characteristics of the initial cohort and after the PSM adjustment. In the initial cohort, the median age was 71 years (64–78). There were more male patients (67%), but the sex distribution was comparable between both groups (*p* = 0.14). Some significant differences were observed between both groups: BMI, ECOG, administration of NAC, CT tumor stage, previous bladder cancer, and follow-up length. After the PSM adjustment, these characteristics were balanced between the two groups, except for follow-up length (see Table 1).

### 3.2. Perioperative Outcomes

The median operative time was 251 min in the overall cohort. Patients undergoing a laparoscopic approach were more likely to have achieved a pentafecta (66% vs. 34% for the open approach; *p* < 0.001). The overall median length of hospital stay was 7 (4–10) days; it was shorter in patients who achieved a pentafecta (5 vs. 8, *p* = <0.001). The overall complication rate was also lower in the pentafecta group (17.7% vs. 40%), and there were also fewer minor complications (CDC I-II) (17.7% vs. 22.8%). After the PSM adjustment, these factors were balanced between both groups, except for the variables included in the definition of the pentafecta, such as the transfusion and complication rates (see Table 2).

### 3.3. Pentafecta Validation

**Figure 1:**
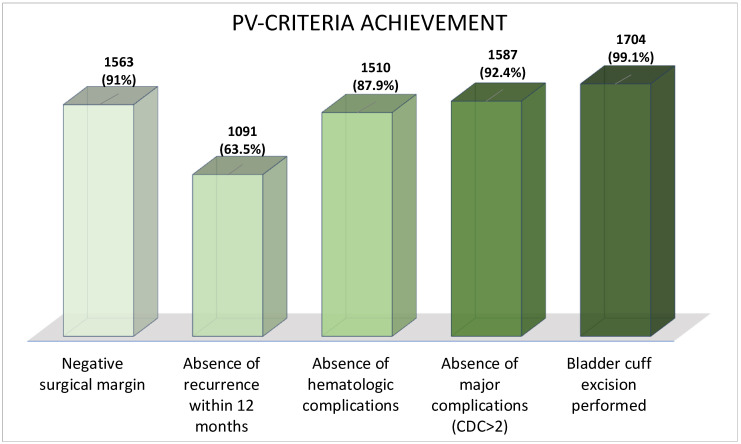
The proportion that achieved each pentafecta criterion in the 1718 patients treated with RNU for high-risk UTUC. CDC = Clavien–Dindo Classification.

In the overall cohort, 844 patients (49%) validated the pentafecta. The proportion that achieved each pentafecta criterion is shown in Figure 1. For intraoperative outcomes, en bloc bladder cuff excision was performed in 1704 patients (99.1%), and negative surgical margins were achieved in 1563 patients (91%). An absence of hematological complications was found in 1510 patients (87.9%), and 1587 patients (92.4%) had no major complications (CDC ≥ 3). The most discriminating criterion of the pentafecta was the absence of any type of recurrence, including bladder recurrence, at 12 months after surgery, which was achieved in only 1091 patients (63.5%) (see Figure 1).

### 3.4. Pathological Characteristics

In the unmatched cohort, the groups showed significant differences with regard to pathological tumor stage, tumor multifocality, lymph node involvement, lymphovascular invasion, concomitant carcinoma in situ, and surgical margins (all *p* < 0.004). All pathological data are summarized in Table 3. After the PSM adjustment, all pathological variables were balanced between the two groups, allowing for a balanced assessment of the oncological outcomes.

### 3.5. Oncological Outcomes

The co-primary endpoint of our study was to evaluate the association of the pentafecta on oncological outcomes. In the PSM-adjusted cohort, the overall median follow-up was 29 (14.0–52.0) months, 32 (15.0–59.0) in the pentafecta group and 26 (13.0–47.0) in the non-pentafecta group, respectively, (*p* < 0.001).

**Figure 2:**
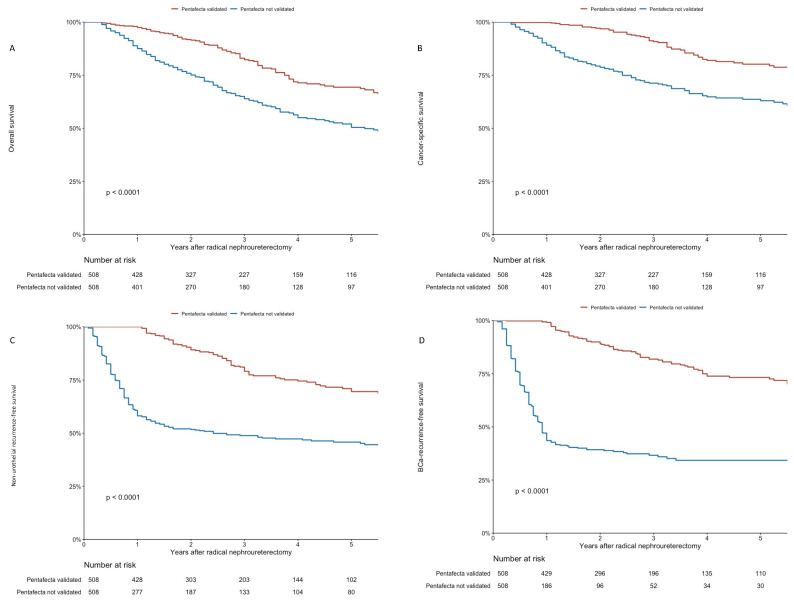
(**A**–**D**): Oncologic outcomes of 1016 patients treated with RNU for high-risk UTUC after propensity-score matching. Kaplan-Meier curves visualizing overall (**A**), cancer-specific (**B**), non-urothelial recurrence-free (**C**), and bladder-cancer recurrence-free survival (**D**).

In our PSM-adjusted cohort, the 5-year estimates for OS were 68.7% (95% CI: 63.5–74.3) for the pentafecta group and 50.1% (95% CI: 44.7–56.2) for the non pentafecta group. Additionally, the 5-year estimates for CSS, NURFS, and BRFS for the pentafecta and non-pentafecta groups were 79.8% (74.9–85.1) vs. 62.7% (57.4–68.5), 70.6% (65.2–76.5) vs. 45.6% (40.9–50.7), and 73.3% (68.1–78.9) vs. 34.3% (29.4–40.0), respectively. OS, CSS, NURFS, and BRFS were significantly higher in the pentafecta group, as visualized in the Kaplan–Meier curves (all *p* < 0.001) (see Figure 2A–D).

PSM-matched multivariable Cox proportional hazard regression analyses that adjusted for the effects of age, sex, ASA, ECOG, BMI, preoperative creatinine, NAC, CT stage and clinical lymph node enlargement, pathological tumor stage and grade, tumor multifocality, concomitant CIS, surgical approach, LND, positive pathological lymph nodes, lymphovascular involvement, and perioperative bladder instillation showed a strong association of the pentafecta with prolonged OS (HR: 0.52, 95% CI: 0.41–0.66; *p* < 0.001), CSS (HR: 0.34, 95% CI: 0.25–0.47; *p* < 0.001), and NURFS (HR: 0.24, 95% CI: 0.19–0.30; *p* < 0.001) (see Table 4). We also assessed the weight of each criterion of the pentafecta on OS; we found that recurrence in the first 12 months had the highest relevance (HR: 2.4, 95% CI: 2.03, 3.86; *p* <0.001), followed by positive surgical margins (HR: 1.9, 95% CI 1.44, 2.39; *p* < 0.001), major complications (HR: 1.8, 95% CI 1.32, 2.33; *p* < 0.001), hematologic complications (HR: 1.4, 95% CI 1.15, 1.84; *p* = 0.004), and the failure to perform an en bloc bladder cuff (HR: 1.7, 95% CI 0.89, 3.15, *p* = 0.61) (see Figure S2). The oncological outcomes of the unmatched cohort are available in Figure S3.

To evaluate our secondary objective regarding preoperative factors that might be able to predict not reaching the pentafecta, we used a logistic regression model. In the PSM-adjusted cohort, no baseline characteristic was found to predict pentafecta failure (all *p* >0.08) (see Table 5).

## 4. Discussion

Nowadays, assessing surgical quality and perioperative management using dedicated tools is of utmost importance for clinicians, institutions, and stakeholders. This is of particular importance to warrant the quality of care in rare diseases, where therapeutic management might become challenging in non-expert centers. However, there were no quality assessment indicators available to practically assess the treatment of HR-UTUC. Therefore, this is the first study to propose a pentafecta to standardize the perioperative management evaluation of RNU in HR-UTUC. Moreover, the achievement of the pentafecta was shown to be correlated with an improvement in the oncological outcomes, making it a great quality indicator of therapeutic success.

Two of the five criteria included in the pentafecta were related to the quality of surgery: achieving negative surgical margins and performing an en bloc bladder cuff excision. Indeed, several studies have shown that positive surgical margins were associated with worse survival outcomes after RNU [3,32,33]. Generally, surgical margins could be avoided by removing the kidney without opening the Gerota’s fascia and avoiding incising the urinary tract or contact between the instruments and the tumor [3]. In our study, the rate of positive surgical margins was 9%, which is in accordance with previous studies of large cohorts [32,34]. For the bladder cuff, a complete resection of the distal ureter and its orifice is mandatory to reduce the risk of local and bladder recurrence [3,8,35,36]. In our study, only 0.9% of cases were performed without an en bloc bladder cuff resection, which confirms that this important surgical step is widely accepted and performed in the expert centers involved in this study [37]. Although performing LND could also be proposed, as it has been recently included in the guidelines, this criterion was not retained due to the heterogeneity of practice and the lack of strong evidence.

In order to cover the whole therapeutic management of the RNU and reflect the safety of the procedure and the best patient care, it was essential to also include morbidity criteria into our pentafecta [12,13,14,38]. Therefore, we included the occurrence of major and hematologic complications. Indeed, hematologic complications are known to be the most common postoperative complications after RNU, and major complications are already recognized as key criteria for the evaluation of perioperative management [39,40]. In our study, 29% of the patients experienced at least one complication of any grade, which is consistent with other reports on RNU [17,40]. Hematologic complications (including thromboembolic events) occurred in 12% of the patients; although, as we were not able to obtain these data, this rate might be minimized by better perioperative management of anticoagulant and antiplatelet therapies [41,42,43].

As a fifth criterion of the pentafecta, we included the absence of tumor recurrence within the first 12 months. Although some can debate the implementation of an oncological outcome into a perioperative assessment tool, it reflects the adequate oncologic management of HR-UTUC patients. The concept of including oncological outcomes next to functional results in a surgical assessment was initially proposed by Salomon et al. [11] and has been implemented in most of the tools used in the other urologic oncology surgeries [12,13,14,38]. Indeed, early RFS could be mostly reduced nowadays when applying appropriate therapeutic management criteria, such as accurate surgical indications, perioperative systemic therapies, and perioperative bladder instillations [20,21,22]. Although there are three important steps of HR-UTUC patient management that may impact the early RFS, due to the constant improvement of the guidelines, it was not possible to precisely assess these steps for every patient in this historical cohort. Therefore, when using early RFS, this reflects oncological management more widely and could also be applied in the most contemporary cohorts.

In this study, the validation of the pentafecta had a great predictive value for OS, CSS, NURFS, and BRFS. Multivariable Cox regression models that adjusted for potential confounders confirmed these associations. Although an oncological benefit might have been expected based on the criteria of the pentafecta, the important and statistically significant improvement of OS with an HR of 0.52 when achieved ensures the quality of this tool regarding its clinical impact. Indeed, the standardization of outcome assessment has the final goal to improve patient survival.

When we sought predictors of failure or validation of the pentafecta, we did not find any preoperative or baseline patient characteristics in the PSM-adjusted cohort. This result suggests that the pentafecta purely reflects the quality of care and perioperative management, without being impacted by the patients themselves. Indeed, it is of utmost importance that these criteria, which are made to assess the quality of care, are not impacted by the patient but only by the therapeutic management. Nevertheless, external validation in a contemporary cohort remains required to confirm its use in daily practice.

Although we have developed an innovative and promising standardization tool to assess the perioperative management of HR-UTUC patients treated by RNU, our study is not exempt from limitations. First, its multicentric and retrospective design may have resulted in various surgical techniques and experiences that may lead to selection bias. Nevertheless, to limit bias coming from missing data and heterogeneous data, we performed multiple imputations and propensity-score matching [31,44]. Second, we were not able to assess surgeon experience and consequently show if the validation of the pentafecta is correlated with the learning curve. Therefore, in order to confirm that the pentafecta could be used as a great representative of quality of care, future studies will have to assess its correlation with surgeon experience and center expertise. Additionally, to reduce bias, techniques such as the performance of an accurate bladder cuff removal should be confirmed by pathological reports in future studies and not primarily by surgeons’ reports. Third, due to its retrospective design, all the actual standards of care (perioperative instillations, lymph node dissection, or systemic therapies) could not always be reflected; consequently, the use of early recurrence as one of the endpoints may better reflect the overall oncological management, as most of the perioperative treatments were shown to impact RFS or CSS. Moreover, some data, such as anticoagulant intake, were not available and would be interesting to assess to limit cofounders regarding hematologic complications. Finally, the main limitation of this study remains in the need for external validation to be used by institutions, stakeholders, and surgeons to assess the therapeutic management of patients with HR-UTUC.

## 5. Conclusions

We proposed for the first time a pentafecta as quality-of-care criteria for the perioperative management of patients undergoing RNU for HR-UTUC. Indeed, establishing quality indicators for UTUC may help to better understand the prognosis and potentially improve patient care. When validated, the pentafecta showed good predictive reliability on oncological outcomes (OS, CSS, BRFS, and NURFS) without being impacted by patients’ characteristics, suggesting it is a good reflection of the perioperative care itself. Therefore, the pentafecta could be used to standardize the evaluation of perioperative care for HR-UTUC. Nevertheless, further studies are needed to externally validate the pentafecta in contemporary cohorts.

## Figures and Tables

**Table 1:** Clinicopathological characteristics of 1718 patients treated with RNU for high-risk UTUC, initial cohort (**left**) and after propensity-score-matching adjustment (**right**), both stratified by pentafecta validation. PSM = propensity-score matching; BMI = Body mass index; ECOG = Eastern Cooperative Oncology Group Status; ASA = American Society of Anesthesiologists Score; NAC = Neoadjuvant chemotherapy; IQR = interquartile range.

Characteristic	Initial Cohort				PSM-Adjusted Cohort			
	Total Cohort	Pentafecta Validation			Total Cohort	Pentafecta Validation		
	*n* = 1718	Pentafecta Validated, *n* = 844 (49%)	Pentafecta Not Validated, *n* = 874 (51%)	*p*-Value	*n* = 1016	Pentafecta Validated, *n* = 508 (50%)	Pentafecta Not Validated, *n* = 508 (50%)	*p*-Value
**Age**	71 (64, 78)	71 (64, 77)	72 (64, 78)	0.12	71 (64, 78)	71 (65, 78)	72 (64, 78)	0.8
**Sex**				0.14				0.8
male	1153 (67%)	552 (65%)	601 (69%)		681 (67%)	339 (67%)	342 (67%)	
female	565 (33%)	292 (35%)	273 (31%)		335 (33%)	169 (33%)	166 (33%)	
**BMI**	26.0 (23.0, 29.0)	26.0 (23.4, 29.0)	25.4 (23.0, 28.7)	0.027	25.8 (23.0, 28.7)	26.0 (23.1, 28.5)	25.7 (22.9, 28.7)	0.6
Unknown	267	106	161		-	-	-	
**ECOG**				0.047				0.8
0	844 (62%)	451 (65%)	393 (58%)		630 (62%)	318 (63%)	312 (61%)	
1	401 (29%)	188 (27%)	213 (32%)		293 (29%)	146 (29%)	147 (29%)	
2	108 (7.9%)	46 (6.6%)	62 (9.2%)		83 (8.2%)	38 (7.5%)	45 (8.9%)	
3	14 (1.0%)	8 (1.2%)	6 (0.9%)		10 (1.0%)	6 (1.2%)	4 (0.8%)	
Unknown	351	151	200		-	-	-	
**Diabetes mellitus**	289 (22%)	152 (21%)	137 (22%)	0.7	202 (20%)	103 (20%)	99 (19%)	0.8
Unknown	378	128	250		-	-	-	
**ASA**				0.2				0.4
1	101 (6.8%)	57 (7.6%)	44 (6.0%)		78 (7.7%)	43 (8.5%)	35 (6.9%)	
2	676 (46%)	343 (46%)	333 (46%)		494 (49%)	247 (49%)	247 (49%)	
3	669 (45%)	338 (45%)	331 (45%)		426 (42%)	212 (42%)	214 (42%)	
4	35 (2.4%)	12 (1.6%)	23 (3.1%)		18 (1.8%)	6 (1.2%)	12 (2.4%)	
Unknown	237	94	143		-	-	-	
**Hypertension**	682 (56%)	354 (54%)	328 (58%)	0.14	575 (56.6%)	271 (26.7%)	304 (29.9%)	0.9
Unknown	505	192	313		-	-	-	
**NAC**	115 (6.7%)	46 (5.5%)	69 (7.9%)	0.043	94 (9.3%)	41 (8.1%)	53 (10%)	0.2
**CT tumor stage**				0.043				
cT0	83 (4.8%)	41 (4.9%)	42 (4.8%)		48 (4.7%)	25 (4.9%)	23 (4.5%)	0.9
cTa/cT1	559 (33%)	297 (35%)	262 (30%)		318 (31%)	162 (32%)	156 (31%)	
cT2	248 (14%)	108 (13%)	140 (16%)		150 (15%)	74 (15%)	76 (15%)	
cT ≥ 3	267 (15.6%)	124 (14.9%)	143 (16.3%)		161 (15.6%)	86 (16.6%)	75 (14.6%)	
cTx (infiltration unclear)	561 (33%)	274 (32%)	287 (33%)		339 (33%)	161 (32%)	178 (35%)	
**CT lymph nodes**				0.005				0.5
cN0	1176 (79%)	604 (82%)	572 (77%)		782 (77%)	397 (78%)	385 (76%)	
lymph nodes < 1 cm	182 (12%)	86 (12%)	96 (13%)		139 (14%)	69 (14%)	70 (14%)	
lymph nodes > 1 cm	122 (8.2%)	44 (6.0%)	78 (10%)		95 (9.4%)	42 (8.3%)	53 (10%)	
**Previous bladder cancer**	510 (31%)	209 (25%)	301 (36%)	<0.001	320 (31%)	158 (31%)	162 (32%)	0.8
Unknown	57	18	39					
**Smoking status**				0.9				0.9
currently smoking	388 (25%)	200 (25%)	188 (24%)		380 (37%)	191 (38%)	189 (37%)	
former smoking	618 (39%)	308 (39%)	310 (40%)		406 (40%)	199 (39%)	207 (41%)	
never smoked	573 (36%)	289 (36%)	284 (36%)		230 (23%)	118 (23%)	112 (22%)	
Unknown	139	47	92		-	-	-	
**Follow-up**	28 (13, 52)	33 (15, 59)	24 (12, 46)	<0.001	29 (14, 52)	32 (15, 59)	26 (13, 47)	<0.001

*n* (%); Median (IQR). Wilcoxon rank sum test; Pearson’s Chi-squared test; Fisher’s exact test.

**Table 2:** Perioperative characteristics of 1718 patients treated with RNU for high-risk UTUC, initial cohort (**left**) and after propensity-score-matching adjustment (**right**), both stratified by pentafecta validation. PSM = propensity-score matching; CDC = Clavien–Dindo Classification; IQR = interquartile range.

Characteristic	Initial Cohort				PSM-Adjusted Cohort			
	Total Cohort	Pentafecta Validation			Total Cohort	Pentafecta Validation		
	*n* = 1718	Pentafecta Validated, *n* = 844 (49%)	Pentafecta Not Validated, *n* = 874 (51%)	*p*-Value	*n* = 1016	Pentafecta Validated, *n* = 508 (50%)	Pentafecta Not Validated, *n* = 508 (50%)	*p*-Value
**Surgical approach**				<0.001				0.9
open	689 (40%)	290 (34%)	399 (46%)		430 (42%)	214 (42%)	216 (43%)	
laparoscopic or robotic	1029 (60%)	554 (66%)	475 (54%)		586 (58%)	294 (58%)	292 (57%)	
**Side**				0.2				0.5
left	838 (50%)	403 (48%)	435 (52%)		496 (49%)	242 (48%)	254 (50%)	
right	840 (50%)	433 (52%)	407 (48%)		520 (51%)	266 (52%)	254 (50%)	
bilateral	0 (0%)	0 (0%)	0 (0%)		0 (0%)	0 (0%)	0 (0%)	
Unknown	40	8	32		-	-	-	
**Transfusion**	153 (9.7%)	0 (0%)	153 (20%)	<0.001	119 (12%)	1 (0.2%)	118 (23%)	<0.001
Unknown	146	26	120		-	-	-	
**Surgery duration**	251 (195, 325)	247 (190, 317)	258 (195, 338)	0.049	247 (190, 320)	245 (192, 316)	250 (187, 328)	0.5
Unknown	677	298	379		-	-	-	
**Lymph node dissection (LND)**	722 (42%)	340 (40%)	382 (44%)	0.2	456 (45%)	222 (44%)	234 (46%)	0.4
**Postoperative instillation**	197 (14%)	98 (14%)	99 (15%)	0.5	176 (17%)	88 (17%)	88 (17%)	0.9
Unknown	358	137	221		-	-	-	
**Hospital length of stay**	7.0 (4.0, 10.0)	5.0 (3.0, 9.0)	8.0 (5.0, 13.0)	<0.001	7.0 (4.0, 10.0)	6.0 (4.0, 9.0)	8.0 (4.0, 12.0)	<0.001
Unknown	309	83	226					
**Complications**				<0.001				<0.001
No complication	1142 (72%)	694 (82%)	448 (60%)		721 (71%)	416 (82%)	305 (60%)	
CDC I	100 (6.3%)	57 (6.8%)	43 (5.8%)		65 (6.4%)	36 (7%)	29 (6%)	
CDC II	216 (14%)	92 (11%)	124 (17%)		142 (14%)	56 (11%)	86 (17%)	
CDC III	82 (5.2%)	0 (0%)	82 (11%)		56 (5.5%)	0 (0%)	56 (11%)	
CDC IV	33 (2.1%)	0 (0%)	33 (4.4%)		22 (2%)	0 (0%)	22 (4%)	
CDC V	15 (0.9%)	0 (0%)	15 (2.0%)		10 (0.9%)	0 (0%)	10 (2%)	

Median (IQR); *n* (%). Wilcoxon rank sum test; Pearson’s Chi-squared test; Fisher’s exact test.

**Table 3:** Pathological characteristics of 1718 patients treated with RNU for high-risk UTUC, initial cohort (**left**) and after propensity-score-matching adjustment (**right**), both stratified by pentafecta validation. PSM = propensity-score matching; IQR = interquartile range.

Characteristic	Initial Cohort				PSM-Adjusted Cohort			
	Total Cohort	Pentafecta Validation			Total Cohort	Pentafecta Validation		
	*n* = 1718	Pentafecta Validated, *n* = 844 (49%)	Pentafecta Not Validated, *n* = 874 (51%)	*p*-Value	*n* = 1016	Pentafecta Validated, *n* = 508 (50%)	Pentafecta Not Validated, *n* = 508 (50%)	*p*-Value
**Pathological tumor stage**				<0.001				0.6
pT0	48 (2.8%)	26 (3.1%)	22 (2.5%)		24 (2.4%)	15 (3.0%)	9 (1.8%)	
pTa	340 (20%)	215 (25%)	125 (14%)		181 (18%)	92 (18%)	89 (18%)	
pTis	89 (5.2%)	48 (5.7%)	41 (4.7%)		41 (4.0%)	20 (3.9%)	21 (4.1%)	
pT1	322 (19%)	168 (20%)	154 (18%)		184 (18%)	95 (19%)	89 (18%)	
pT2	271 (16%)	125 (15%)	146 (17%)		173 (17%)	83 (16%)	90 (18%)	
pT3	581 (34%)	248 (29%)	333 (38%)		375 (37%)	189 (37%)	186 (37%)	
pT4	67 (3.9%)	14 (1.7%)	53 (6.1%)		38 (3.7%)	14 (2.8%)	24 (4.7%)	
**Pathological tumor grade**				0.074				0.4
no tumor	24 (1.4%)	13 (1.5%)	11 (1.3%)		15 (1.5%)	8 (1.6%)	7 (1.4%)	
Low-grade	326 (19%)	178 (21%)	148 (17%)		191 (19%)	101 (20%)	90 (18%)	
High-grade	1368 (80%)	653 (77%)	715 (82%)		810 (81%)	399 (80%)	411 (82%)	
**Multifocal**	704 (41%)	299 (36%)	405 (47%)	<0.001	396 (39%)	199 (39%)	197 (39%)	0.9
Unknown	9	4	5		-	-	-	
**Lymph node involvement**				<0.001				0.3
no	561 (33%)	288 (34%)	273 (31%)		319 (31%)	162 (32%)	157 (31%)	
yes	214 (12%)	73 (8.6%)	141 (16%)		148 (15%)	65 (13%)	83 (16%)	
Nx (No LND)	943 (55%)	483 (57%)	460 (53%)		549 (54%)	281 (55%)	268 (53%)	
**Lymphovascular invasion**	330 (19%)	121 (14%)	209 (24%)	<0.001	214 (21%)	101 (20%)	113 (22%)	0.4
**Concomitant carcinoma in situ**	313 (18%)	131 (16%)	182 (21%)	0.004	173 (17%)	84 (17%)	89 (18%)	0.7

Median (IQR); *n* (%). Wilcoxon rank sum test; Pearson’s Chi-squared test; Fisher’s exact test.

**Table 4:** Uni- and multivariable Cox regression analyses assessing the association of a validation of the pentafecta with non-urothelial recurrence-free, cancer-specific, and overall survival. PNV = Pentafecta not validated; ASA = American Society of Anesthesiologists Score; ECOG = Eastern Cooperative Oncology Group Status; BMI = Body mass index; NAC = neoadjuvant chemotherapy; LND = lymph node dissection.

Characteristic	Overall Survival						Cancer-Specific Survival						Non-Urothelial Recurrence-Free Survival					
	Univariable			Multivariable			Univariable			Multivariable			Univariable			Multivariable		
	HR	95% CI	*p*-Value	HR	95% CI	*p*-Value	HR	95% CI	*p*-Value	HR	95% CI	*p*-Value	HR	95% CI	*p*-Value	HR	95% CI	*p*-Value
**Pentafecta validation (Reference: PNV)**																		
Pentafecta validated	0.54	0.43, 0.68	<0.001	0.52	0.41, 0.66	<0.001	0.37	0.27, 0.50	<0.001	0.34	0.25, 0.47	<0.001	0.28	0.22, 0.35	<0.001	0.24	0.19, 0.30	<0.001
**Age**	1.03	1.01, 1.04	<0.001	1.02	1.00, 1.03	0.016	1.00	0.99, 1.02	0.604	1.00	0.99, 1.02	0.7	1.01	1.00, 102	0.295	1.00	0.99, 1.02	0.4
**Sex (Reference: Male)**																		
female	0.88	0.69, 1.12	0.308	0.85	0.66, 1.10	0.2	1.05	0.79, 1.41	0.716	1.07	0.78, 1.46	0.7	1.08	0.87, 1.34	0.484	0.96	0.76, 1.21	0.7
**ASA**	1.43	1.20, 1.72	<0.001	1.31	1.04, 1.66	0.023	1.18	0.95, 1.47	0.135	1.13	0.85, 1.51	0.4	1.16	0.98, 1.37	0.076	1.15	0.93, 1.41	0.2
**ECOG**	1.40	1.21, 1.63	<0.001	1.09	0.92, 1.28	0.3	1.51	1.27, 1.80	<0.001	1.20	0.98, 1.47	0.083	1.23	1.07, 1.42	0.004	1.09	0.94, 1.28	0.3
**BMI**	0.99	0.97, 1.01	0.427	0.97	0.95, 1.00	0.036	0.99	0.96, 1.01	0.331	0.98	0.95, 1.01	0.2	1.00	0.98, 1.03	0.661	1.01	0.99, 1.03	0.4
**Preoperative creatinine**	1.13	1.03, 1.23	0.008	1.03	0.89, 1.19	0.7	1.11	0.99, 1.24	0.076	1.00	0.84, 1.20	0.9	0.97	0.86, 1.10	0.657	0.78	0.64, 0.94	0.010
**NAC (Reference: No)**																		
yes	1.25	0.86, 1.83	0.243	0.87	0.56, 1.35	0.5	1.83	1.23, 2.73	0.003	1.10	0.68, 1.78	0.7	1.75	1.28, 2.38	<0.001	1.02	0.70, 1.48	0.9
**CT stage (Reference: cT0)**																		
cTa/cT1	0.74	0.56, 0.97	0.027	0.85	0.63, 1.15	0.3	0.71	0.50, 1.00	0.049	0.73	0.50, 1.08	0.12	1.01	0.78, 1.30	0.945	1.05	0.79, 1.39	0.7
cT2	0.59	0.39, 0.87	0.008	0.56	0.36, 0.86	0.008	0.74	0.47, 1.16	0.188	0.53	0.32, 0.90	0.018	0.99	0.71, 1.37	0.945	0.88	0.61, 1.27	0.5
≥cT3	1.13	0.83, 1.54	0.441	0.99	0.70, 1.40	0.9	1.24	0.85, 1.81	0.259	0.94	0.62, 1.42	0.8	1.46	1.08, 1.95	0.013	1.30	0.95, 1.80	0.10
**CT lymph nodes (Reference: cN0)**																		
Lymphnodes < 1 cm	1.19	0.87, 1.63	0.277	1.03	0.72, 1.47	0.9	1.45	1.00, 2.10	0.052	1.13	0.73, 1.74	0.6	1.41	1.06, 1.87	0.017	1.14	0.82, 1.57	0.4
Lymphnodes > 1 cm	2.17	1.59, 2.95	<0.001	1.55	1.06, 2.28	0.024	2.80	1.96, 3.99	<0.001	1.57	1.00, 2.48	0.052	2.59	1.94, 3.45	<0.001	2.24	1.56, 3.23	<0.001
**CT hydronephrosis**	0.93	0.74, 1.16	0.501	1.06	0.83, 1.35	0.6	1.04	0.79, 1.37	0.783	1.25	0.93, 1.68	0.15	0.93	0.75, 1.14	0.476	0.96	0.77, 1.19	0.7
**Pathological tumor stage (Reference: pT0)**																		
pTa	1.20	0.29, 4.97	0.802	0.65	0.08, 5.03	0.7	0.57	0.13, 2.47	0.456	0.66	0.08, 5.62	0.7	0.42	0.18, 1.01	0.053	0.80	0.18, 3.57	0.8
pTis	1.21	0.26, 5.52	0.808	0.60	0.07, 5.05	0.6	0.77	0.16, 3.83	0.752	0.93	0.09, 9.08	0.9	0.78	0.30, 2.03	0.605	1.15	0.24, 5.55	0.9
pT1	1.42	0.34, 5.83	0.631	0.78	0.10, 5.97	0.8	0.95	0.23, 3.99	0.948	1.17	0.14, 9.74	0.9	0.71	0.31, 1.66	0.431	1.28	0.29, 5.65	0.7
pT2	1.62	0.39, 6.70	0.502	1.14	0.15, 8.70	0.9	1.06	0.25, 4.47	0.932	1.35	0.16, 11.1	0.8	0.94	0.40, 2.17	0.876	1.41	0.32, 6.18	0.6
pT3	2.88	0.71, 11.63	0.138	1.58	0.21, 11.9	0.7	2.08	0.51, 8.42	0.307	2.06	0.26, 16.6	0.5	1.46	0.65, 3.30	0.363	2.07	0.48, 8.94	0.3
pT4	5.94	1.39, 25.42	0.0163	3.36	0.42, 26.6	0.3	4.80	1.10, 20.89	0.037	4.41	0.52, 37.3	0.2	3.30	1.35, 8.07	0.009	4.08	0.90, 18.6	0.069
**Pathological tumor grade (Reference: Low-grade)**																		
High-grade	1.84	1.33, 2.53	<0.001	1.31	0.90, 1.92	0.2	2.71	1.71, 4.30	<0.001	1.70	0.98, 2.93	0.058	2.04	1.48, 2.81	<0.001	1.56	1.08, 2.26	0.019
**Concomitant carcinoma in situ (Reference: No)**																		
yes	1.02	0.77, 1.37	0.881	0.91	0.65, 1.27	0.6	1.11	0.78, 1.57	0.569	0.82	0.54, 1.24	0.4	1.28	0.99, 1.66	0.055	0.99	0.74, 1.34	0.9
**Multifocal tumor (Reference: No)**																		
yes	1.29	1.03, 1.62	0.024	1.48	1.14, 1.91	0.003	1.38	1.05, 1.82	0.022	1.59	1.15, 2.19	0.005	1.09	0.88, 1.35	0.42	1.23	0.98, 1.56	0.080
**Surgical approach (Reference: Open)**																		
Laparoscopic or robotic	0.77	0.62, 0.97	0.024	0.81	0.64, 1.04	0.093	0.64	0.49, 0.85	0.002	0.75	0.55, 1.02	0.067	0.89	0.72, 1.10	0.267	0.96	0.77, 1.21	0.7
**LND (Reference: No)**																		
Yes	1.05	0.84, 1.32	0.651	0.68	0.37, 1.27	0.2	1.52	1.15, 2.00	0.003	1.03	0.45, 2.37	0.9	1.33	1.08, 1.63	0.008	0.73	0.42, 1.28	0.3
**Lymph node involvement (Reference: No)**																		
yes	2.86	2.03, 4.01	<0.001	1.97	1.33, 2.93	<0.001	3.65	2.48, 5.37	<0.001	2.18	1.39, 3.41	<0.001	3.17	2.35, 4.27	<0.001	1.70	1.19, 2.43	0.004
Nx	1.32	1.00, 1.73	0.047	0.96	0.52, 1.77	0.9	1.11	0.79, 1.56	0.549	1.23	0.53, 2.82	0.6	1.13	0.88, 1.46	0.335	0.92	0.53, 1.59	0.8
**Lymphovascular invasion (Reference: No)**																		
yes	1.44	1.10, 1.89	0.007	0.96	0.71, 1.29	0.8	1.86	1.37, 2.54	<0.001	1.15	0.82, 1.62	0.4	2.21	1.76, 2.78	<0.001	1.50	1.16, 1.94	0.002
**Previous bladder cancer (Reference: No)**																		
yes	1.17	0.92, 1.47	0.198	1.20	0.93, 1.54	0.2	1.22	0.91, 1.62	0.181	1.59	1.16, 2.18	0.004	0.99	0.79, 1.24	0.939	1.10	0.86, 1.40	0.5
**Postoperative instillation (Reference: No)**																		
Yes	0.79	0.57, 1.09	0.154	0.66	0.46, 0.97	0.032	0.61	0.39, 0.96	0.031	0.48	0.28, 0.83	0.009	0.98	0.74, 1.29	0.887	0.80	0.58, 1.09	0.15

HR = Hazard Ratio, CI = Confidence Interval.

**Table 5:** Logistic regression analysis assessing predictors for a failure to achieve a pentafecta in patients treated with RNU for high-risk UTUC. ASA = American Society of Anesthesiologists Score; ECOG = Eastern Cooperative Oncology Group Status; BMI = Body mass index; LND = lymph node dissection.

Characteristic	OR	95% CI	*p*-Value
**Age**	1.00	0.99, 1.01	0.9
**Gender (Ref.: Male)**			
female	0.97	0.75, 1.26	0.8
**ASA**	1.11	0.92, 1.34	0.3
**ECOG**	1.04	0.87, 1.24	0.7
**BMI**	1.00	0.98, 1.03	0.9
**Smoking status (Ref.: never)**			
former smoking	1.05	0.79, 1.39	0.7
currently smoking	0.96	0.69, 1.33	0.8
**Multifocal (Ref.: No)**			
yes	0.98	0.76, 1.27	0.9
**Diabetes mellitus (Ref.: No)**			
yes	0.95	0.70, 1.30	0.8
**Preoperative creatinine**	1.09	0.96, 1.26	0.2
**CT stage (Ref.: cT0)**			
cT1	0.89	0.66, 1.20	0.4
cT2	0.95	0.65, 1.39	0.8
≥cT3	0.81	0.56, 1.17	0.3
**CT lymph nodes (Ref.: cT0)**			
Lymphnodes < 1 cm	1.06	0.74, 1.53	0.7
Lymphnodes > 1 cm	1.45	0.96, 2.20	0.08
**CT hydronephrosis (Ref.: No)**			
Yes	1.10	0.86, 1.41	0.5
**Surgical approach (Ref.: Open)**			
laparoscopic or robotic	0.98	0.77, 1.26	0.9
**Tumor side (Ref.: left)**			
right	0.91	0.71, 1.16	0.5
**LAD (Ref.: No)**			
Yes	1.08	0.85, 1.39	0.5
**Postoperative instillation (Ref.: No)**			
Yes	0.97	0.70, 1.35	0.9

OR = Odds Ratio, CI = Confidence Interval.

## Data Availability

The data presented in this study are available on request from the corresponding author. The data are not publicly available due to privacy restrictions.

## Supplementary Materials

The following are available online at https://www.mdpi.com/article/10.3390/cancers14071781/s1, Figure S1 (A): Absolute mean differences of covariates before (red line) and after (green line) propensity-score weighting. Threshold set at 0.1. (B): Standardized mean differences of covariates before (red dots) and after (green dots) propensity-score weighting. Threshold set at 0.1. (C): Imputed variables for Cox proportional hazard regression analyses. (D): Imputed variables for logistic regression analyses; Figure S2: Weight of the particular pentafecta criteria on the overall survival of 1016 propensity-score-matched patients treated with RNU for high-risk UTUC. (HR = Hazard ratio; CI = confidence interval); Figure S3: Oncologic outcomes of 1718 patients treated with RNU for high-risk UTUC. Kaplan–Meier curves visualizing overall (A), cancer-specific (B), non-urothelial recurrence-free (C), and bladder-cancer recurrence-free survival (D); File S1: Performance of the literature search and consensus survey leading to the selection of the pentafecta criteria.
