# Supplementary material for: Pentafecta for Radical Nephroureterectomy in Patients with High-Risk Upper Tract Urothelial Carcinoma: A Proposal for Standardization of Quality Care Metrics
Source: Cancers (Basel). 2022 Mar 31;14(7):1781. doi: 10.3390/cancers14071781 (PMC8997024; doi:10.3390/cancers14071781)
Supplement: Supplementary file 1 [file cancers-14-01781-s001.zip › cancers-1646661-supplementary/cancers-1646661-supplementary-done.pdf]

cancers

A

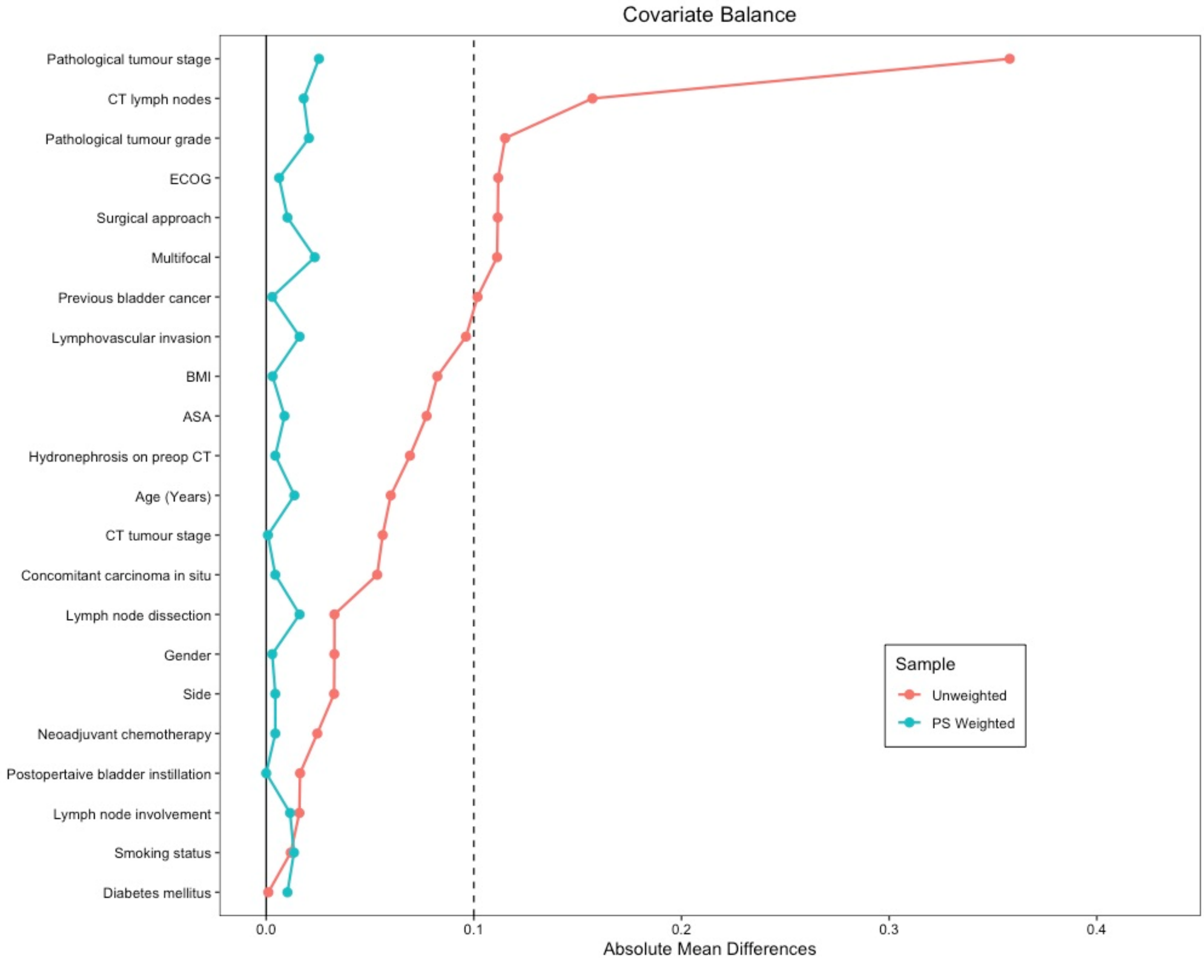
Covariate Balance
Pathological tumour stage
CT lymph nodes
Pathological tumour grade
ECOG
Surgical approach
Multifocal
Previous bladder cancer
Lymphovascular invasion
BMI
ASA
Hydronephrosis on preop CT
Age (Years)
CT tumour stage
Concomitant carcinoma in situ
Lymph node dissection
Gender
Side
Neoadjuvant chemotherapy
Postopertaive bladder instillation
Lymph node involvement
Smoking status
Diabetes mellitus
0.0
0.1
0.2
0.3
0.4
Absolute Mean Differences
Sample
Unweighted
PS Weighted

B

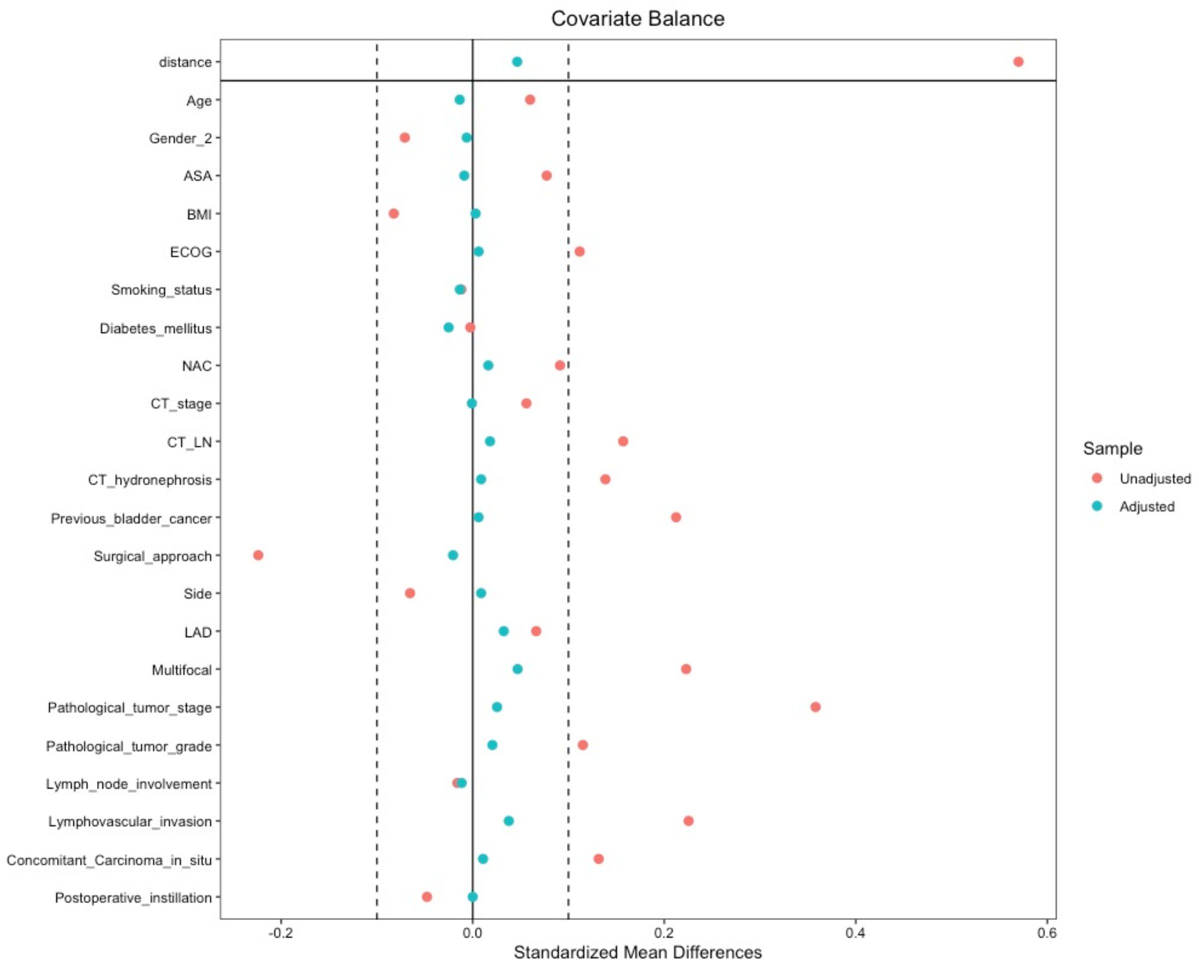

C

Variable		Observations per m		
	Complete	Incomplete	Imputed	Total
**ASA**	1481 (86.2%)	237 (13.8%)	237	1718
**BMI**	1451 (84.5%)	267 (15.5%)	267	1718
**ECOG**	1367 (79.6%)	351 (20.4%)	351	1718
**Preoperative creatinine**	1548 (90.1%)	170 (9.9%)	170	1718
**CT lymph nodes**	1480 (86.1%)	238 (13.9%)	238	1718
**CT hydronephrosis**	1538 (89.5%)	180 (10.5%)	180	1718
**Previous bladder cancer**	1661 96.7%)	57 (3.3%)	57	1718
**Multifocality**	1709 (99.5%)	9 (0.5%)	9	1718
**Postoperative instillation**	1360 (79.2%)	358 (20.8%)	358	1718

D

Variable		Observations per m		
	Complete	Incomplete	Imputed	Total
**ASA**	1481 (86.2%)	237 (13.8%)	237	1718
**BMI**	1451 (84.5%)	267 (15.5%)	267	1718
**ECOG**	1367 (79.6%)	351 (20.4%)	351	1718
**Smoking status**	1579 (91.9%)	139 (8.1%)	139	1718
**Diabetes mellitus**	1340 (78.0%)	378 (22.0%)	378	1718
**Preoperative creatinine**	1548 (90.1%)	170 (9.9%)	170	1718
**CT lymph nodes**	1480 (86.1%)	238 (13.9%)	238	1718
**CT hydronephrosis**	1538 (89.5%)	180 (10.5%)	180	1718
**Previous bladder cancer**	1661 96.7%)	57 (3.3%)	57	1718
**Tumour side**	1678 (97.7%)	40 (2.3%)	40	1718
**Multifocality**	1709 (99.5%)	9 (0.5%)	9	1718
**Postoperative instillation**	1360 (79.2%)	358 (20.8%)	358	1718
**Transfusion**	1572 (91.5%)	146 (8.5%)	146	1718

Figure S1. (A): Absolute mean differences of covariates before (red line) and after (green line) propensity score weighting. Threshold set at 0.1. **(B)**: Standardized mean differences of covariates before (red dots) and after (green dots) propensity score weighting. Threshold set at 0.1. **(C)**: Imputed variables for Cox proportional hazard regression analyses. **(D)**: Imputed variables for logistic regression analyses.

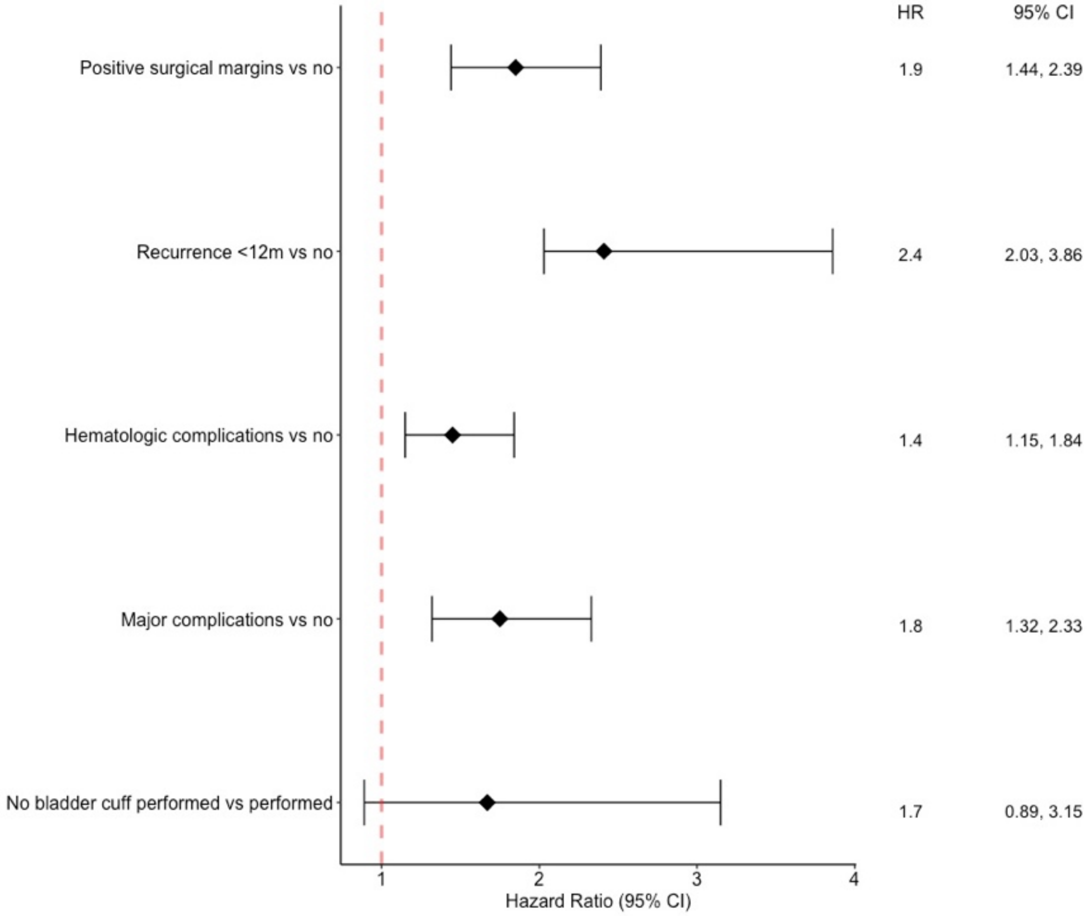

Figure S2. Weight of the particular pentafecta criteria on the overall survival of 1,016 propensity-score matched patients treated with RNU for high-risk UTUC. (*HR=Hazard ratio; CI= confidence interval*).

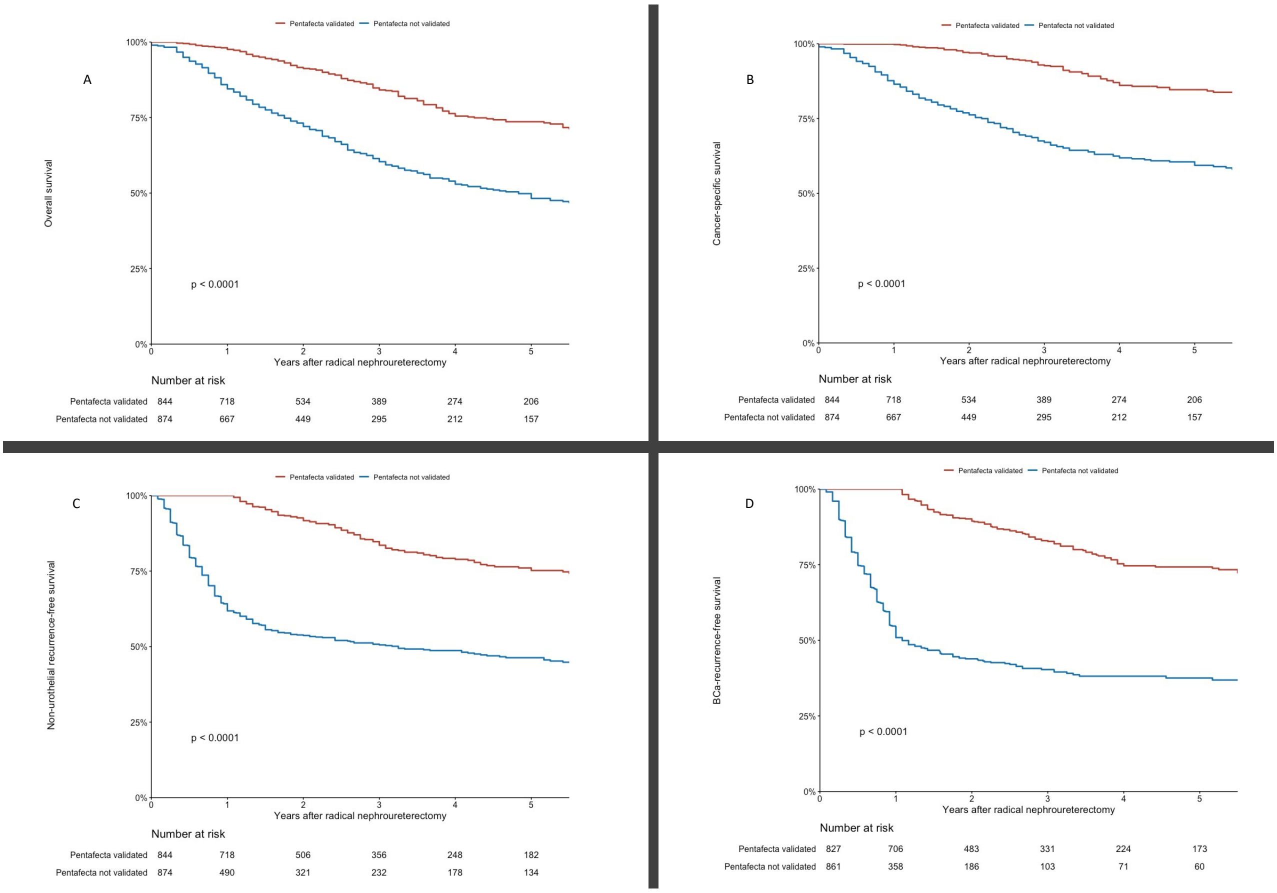

Figure S3. Oncologic outcomes of 1,718 patients treated with RNU for high-risk UTUC. Kaplan-Meier curves visualizing overall **(A)**, cancer-specific **(B)**, non-urothelial recurrence-free **(C)** and bladder-cancer recurrence-free survival **(D)**.
