# Supplementary material for: Pentafecta for Radical Nephroureterectomy in Patients with High-Risk Upper Tract Urothelial Carcinoma: A Proposal for Standardization of Quality Care Metrics
Source: Cancers (Basel). 2022 Mar 31;14(7):1781. doi: 10.3390/cancers14071781 (PMC8997024; doi:10.3390/cancers14071781)
Supplement: Supplementary file 1 [file cancers-14-01781-s001.zip › cancers-1646661-supplementary/Supplementary File S1.pdf]

Supplementary File S1:

Performance of the literature search and consensus survey leading to the selection of the pentafecta criteria.

In order to minimize bias and ensure the best possible validity of the factors selected to reflect the pentafecta, the selection process followed established methods from the field of quality measurement and was done in a stepwise fashion[1]. First, we performed a comprehensive review of the latest literature to extract the most accurate indicators that represent the best evidence available for optimized care of patients with high-risk upper tract urothelial cancer treated with radical nephroureterectomy. We included all relevant articles in English available in the MEDLINE/ Pubmed database up to November 2021. Search terms included “nephroureterectomy”, “upper tract urothelial cancer”, “outcome”, “management”, “quality”, and associated terms. Additionally, guidelines of the relevant urological societies and references of the selected articles were reviewed. By this, relevant factors associated with optimal treatment outcomes were identified. Afterwards, a consensus survey was performed among a panel of urologic and medical oncologists to review and prioritize potential indicators. A final set of five factors that received the highest agreement among the panel members was implemented to create the pentafecta.

1. Wood L, Bjarnason GA, Black PC, Cagiannos I, Heng DY, Kapoor A, et al. Using the Delphi technique to improve clinical outcomes through the development of quality indicators in renal cell carcinoma. J Oncol Pract. 2013;9(5):e262-7.
